# Crystal structure of (4*Z*)-4-{[(2-chloro­phen­yl)amino](furan-2-yl)methyl­idene}-3-methyl-1-phenyl-4,5-di­hydro-1*H*-pyrazol-5-one

**DOI:** 10.1107/S2056989015002698

**Published:** 2015-02-13

**Authors:** Heng-Qiang Zhang, Xing Yang, Qiong Wu, Hong-Li Chen

**Affiliations:** a Department of Chemistry, Hebei Normal University for Nationalities, Chengde, 067000, People’s Republic of China

**Keywords:** crystal structure, pyrazolone, 4-acyl­pyrazolone, *o*-chloro­aniline, hydrogen bonding

## Abstract

In the title compound, C_21_H_16_ClN_3_O_2_, the pyrazolone ring and the O=C—C=C—N mean plane [maximum deviation = 0.022 (2) Å] are nearly coplanar, making a dihedral angle 4.56 (8)°, while the phenyl and pyrazole rings subtend a dihedral angle of 19.75 (8)°. The compound is in the enamine–keto form and its structure is stabilized by an intra­molecular N—H⋯O hydrogen bond. In the crystal, mol­ecules are linked *via* C—H⋯N hydrogen bonds, forming chains along [010]. Between the chains there are π–π inter­actions [inter-centroid distances = 3.3902 (9) and 3.5956 (11) Å], linking the chains to form sheets parallel to (10-1).

## Related literature   

For details of the synthesis of 4-heterocyclic acylpyrazolones, see: Jensen (1959 ▸); Dong *et al.* (1983 ▸). For applications of 4-pyrazolo­nes, see: Casas *et al.* (2007 ▸). For the anti­bacterial activity of pyrazolone derivatives, see: Li *et al.* (2000 ▸); Zhang *et al.* (2008 ▸); Raman *et al.* (2001 ▸). For related structures, see: Zhang *et al.* (2007 ▸); Li *et al.* (2009 ▸).
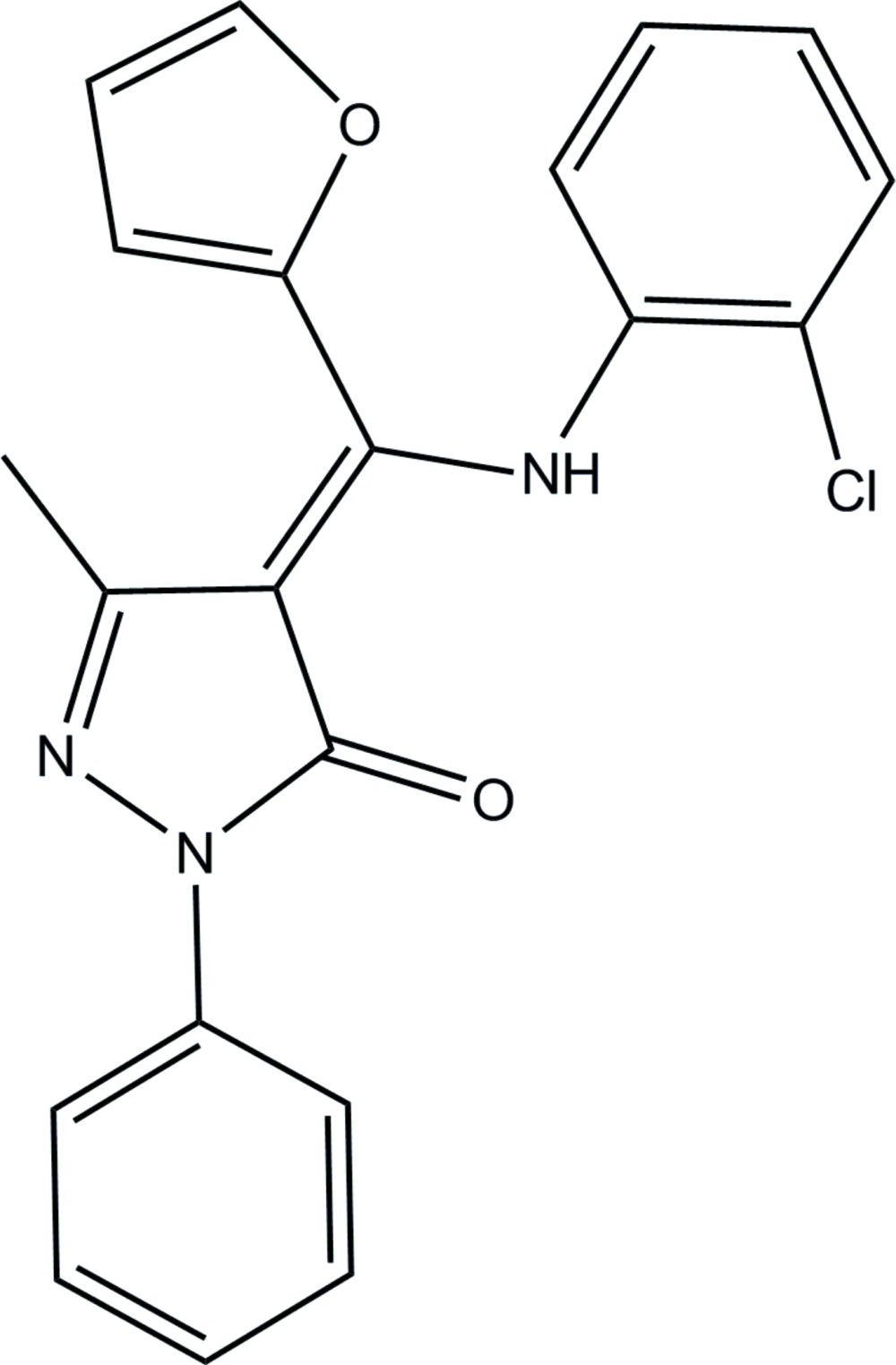


## Experimental   

### Crystal data  

C_21_H_16_ClN_3_O_2_
*M* *_r_* = 377.82
Monoclinic, 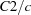

*a* = 17.1008 (16) Å
*b* = 12.4737 (12) Å
*c* = 17.9070 (17) Å
β = 111.276 (2)°
*V* = 3559.4 (6) Å^3^
*Z* = 8
Mo *K*α radiation
μ = 0.24 mm^−1^
*T* = 295 K
0.28 × 0.25 × 0.21 mm

### Data collection  

Bruker APEXII CCD area-detector diffractometer
11255 measured reflections
4048 independent reflections
3245 reflections with *I* > 2σ(*I*)
*R*_int_ = 0.025

### Refinement  

*R*[*F* ^2^ > 2σ(*F* ^2^)] = 0.036
*wR*(*F* ^2^) = 0.095
*S* = 1.01
4048 reflections
245 parameters
H-atom parameters constrained
Δρ_max_ = 0.30 e Å^−3^
Δρ_min_ = −0.33 e Å^−3^

### 

Data collection: *APEX2* (Bruker, 2007 ▸); cell refinement: *SAINT* (Bruker, 2007 ▸); data reduction: *SAINT*; program(s) used to solve structure: *SHELXS97* (Sheldrick, 2008 ▸); program(s) used to refine structure: *SHELXL97* (Sheldrick, 2008 ▸); molecular graphics: *ORTEP-3 for Windows* (Farrugia, 2012 ▸ and *PLATON* (Spek, 2009 ▸); software used to prepare material for publication: *WinGX* publication routines (Farrugia, 2012 ▸) and *PLATON*.

## Supplementary Material

Crystal structure: contains datablock(s) I, New_Global_Publ_Block. DOI: 10.1107/S2056989015002698/su5081sup1.cif

Structure factors: contains datablock(s) I. DOI: 10.1107/S2056989015002698/su5081Isup2.hkl

Supporting information file. DOI: 10.1107/S2056989015002698/su5081Isup3.cml

**Fig. 1.**
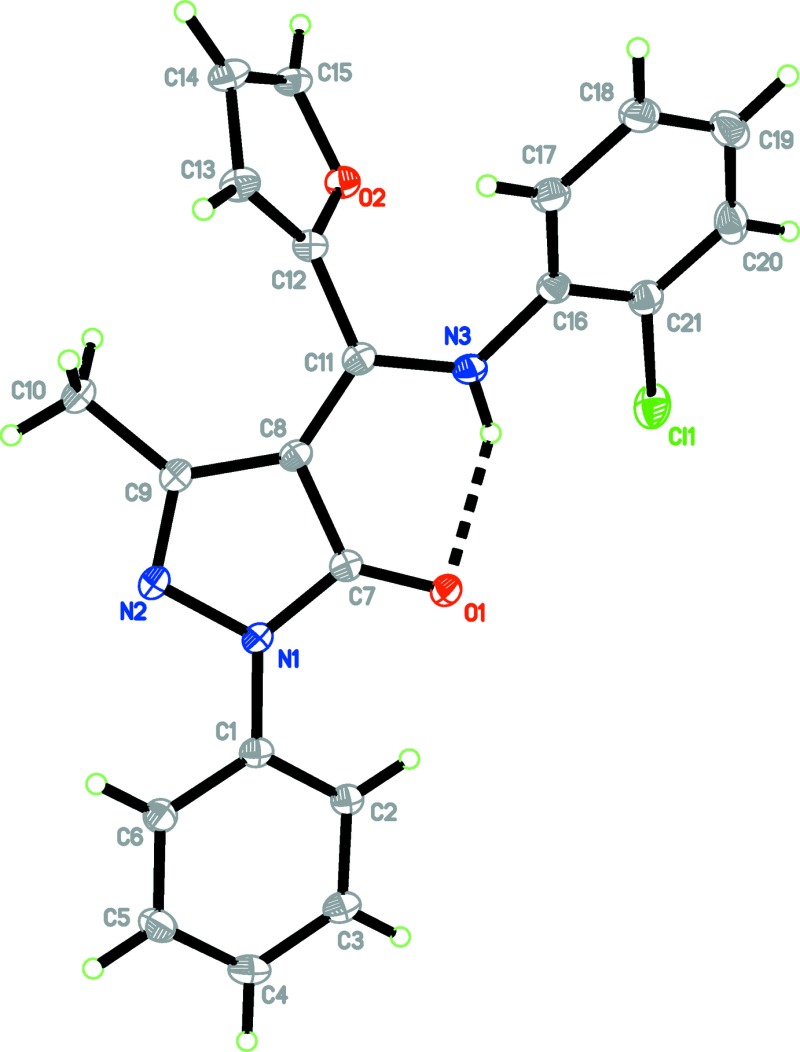
The molecular structure of the title compound, with atom labelling. Displacement ellipsoids are drawn at 30% probability level.

**Fig. 2.**
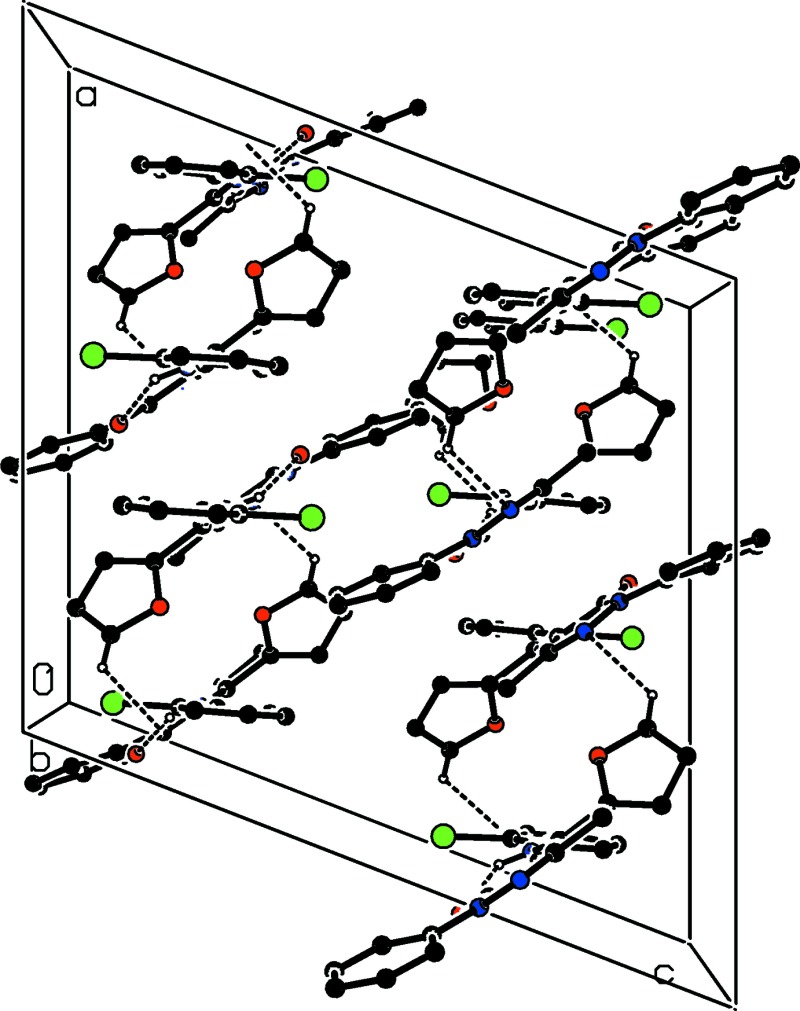
A perspective view along the b axis of the crystal packing of the title compound. The hydrogen bonds are shown as dashed lines (see Table 1 for details; H atoms not involved in hydrogen bonding have been omitted for clarity).

. DOI: 10.1107/S2056989015002698/su5081fig1.tif
The mol­ecular structure of the title compound, with atom labelling. Displacement ellipsoids are drawn at 30% probability level.

b . DOI: 10.1107/S2056989015002698/su5081fig2.tif
A perspective view along the *b* axis of the crystal packing of the title compound. The hydrogen bonds are shown as dashed lines (see Table 1 for details; H atoms not involved in hydrogen bonding have been omitted for clarity).

CCDC reference: 1048268

Additional supporting information:  crystallographic information; 3D view; checkCIF report

## Figures and Tables

**Table 1:** Hydrogen-bond geometry (, )

*D*H*A*	*D*H	H*A*	*D* *A*	*D*H*A*
N3H3*A*O1	0.86	2.00	2.678(2)	135
C15H15N2^i^	0.93	2.59	3.282(2)	131

Symmetry code: (i) 
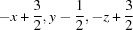
.

## supplementary crystallographic information

### S1. Comment

Pyrazolone derivatives, especially 4-acylpyrazolone, form an important class of organic compounds and represent a significant scientific and applied interest in biological, analytic applications, catalysis, dye and extraction metallurgy (Raman *et al.*, 2001; Casas, *et al.*, 2007). 1-phenyl-3-methyl-4-(2-furoyl)-5-pyrazolone (HPMFP), is a member of a family of 4-heterocyclic acylpyrazolones, first synthesized in 1983 (Dong *et al.*, 1983). In recent years, we have reported on Schiff bases derived from HPMFP and their complexes, which possess high antibacterial activity (Li *et al.*, 2000; Zhang *et al.*, 2008). In order to further investigate the coordination abilities and the behaviour of pyrazolone based ligands, we extended the study to the syntheses of new title pyrazolone derivative, and report herein on its crystal structure.

The molecular structure of the title compound is shown in Fig. 1. The phenyl ring (C1-C6) is twisted by 19.75 (4)° with respect to a plane defined by the pyrazole ring (N1/N2/C7-C9). The pyrazole ring and the (O1/C7/C8/C11/N3) mean plane [maximum deviation = 0.022 (2) Å for atom C7] are nearly coplanar with a dihedral angle 4.56 (8) °. The bond length C8═C11 (1.384 (2) Å) lies between the usual C—C and C═C bond lengths and indicates the delocalization of the electrons because of the addition of a proton to atom N3 which is more favorable than to O1, as shown in the difference Fourier map. Atoms O1 and N3 are on the same side of the C8═C11 bond, hence available for complexation with metals. A strong intramolecular hydrogen bond N3—H3A···O1 (Fig. 1 and Table 1) is also indicative of the enamine-keto form. All bond lengths and angles are normal and comparable with those found for related compounds (Zhang *et al.*, 2007; Li *et al.*, 2009).

In the crystal, molecules are linked via C-H···N hydrogen bonds forming chains along [010]; see Table 1 and Fig. 2. Between the chains there are π-π interactions linking the chains to form sheets parallel to (101) [inter-centroid distances are Cg2···Cg2^i^ = 3.3902 (9) Å and Cg4···Cg4^i^ = 3.5956 (11) Å; Cg2 and Cg4 are the centroids of rings N1/N2/C7-C9 and C16-C21, respectively; symmetry code: (i) -x+1, -y, -z+1].

### S2. Experimental

The starting compound HPMFP was synthesized according to the method proposed by Jensen (1959). A mixture of a 10 ml HPMFP (2 mmol, 0.5366 g) anhydrous ethanol solution, and a 0.21 ml of an *o*-chloroaniline (2 mmol, 0.2545 g) solution was refluxed for *ca*. 5 h, adding a few drops of glacial acetic acid as a catalyst. Then ethanol was removed by evaporation and the resulting black precipitate formed was filtered off, washed with cold anhydrous ethanol and dried in air. Yellow block-like crystals were obtained by slow evaporation of a solution in anhydrous ethanol at room temperature after a few days.

### S3. Refinement

The H atom bonded to N3 was located in a difference Fourier map and freely refined. The C-bound H atoms were placed in calculated positions and refined as riding: C—H = 0.93 - 0.97 Å with *U*_iso_(H) = 1.5_eq_U(C) for methyl H atoms and = 1.2_eq_(C) for other H atoms.

### Crystal data

**Table:**

C_21_H_16_ClN_3_O_2_	*F*(000) = 1568
*M**_r_* = 377.82	*D*_x_ = 1.410 Mg m^−^^3^
Monoclinic, *C*2/*c*	Mo *K*α radiation, λ = 0.71073 Å
Hall symbol: -C 2yc	Cell parameters from 3518 reflections
*a* = 17.1008 (16) Å	θ = 2.6–27.3°
*b* = 12.4737 (12) Å	µ = 0.24 mm^−^^1^
*c* = 17.9070 (17) Å	*T* = 295 K
β = 111.276 (2)°	Block, yellow
*V* = 3559.4 (6) Å^3^	0.28 × 0.25 × 0.21 mm
*Z* = 8	

### Data collection

**Table:**

Bruker APEXII CCD area-detector diffractometer	3245 reflections with *I* > 2σ(*I*)
Radiation source: fine-focus sealed tube	*R*_int_ = 0.025
Graphite monochromator	θ_max_ = 27.4°, θ_min_ = 2.1°
phi and ω scans	*h* = −22→22
11255 measured reflections	*k* = −16→16
4048 independent reflections	*l* = −11→23

### Refinement

**Table:**

Refinement on *F*^2^	Primary atom site location: structure-invariant direct methods
Least-squares matrix: full	Secondary atom site location: difference Fourier map
*R*[*F*^2^ > 2σ(*F*^2^)] = 0.036	Hydrogen site location: inferred from neighbouring sites
*wR*(*F*^2^) = 0.095	H-atom parameters constrained
*S* = 1.01	*w* = 1/[σ^2^(*F*_o_^2^) + (0.0422*P*)^2^ + 2.6702*P*] where *P* = (*F*_o_^2^ + 2*F*_c_^2^)/3
4048 reflections	(Δ/σ)_max_ = 0.001
245 parameters	Δρ_max_ = 0.30 e Å^−^^3^
0 restraints	Δρ_min_ = −0.33 e Å^−^^3^

### Special details

**Table:**

Geometry. All e.s.d.'s (except the e.s.d. in the dihedral angle between two l.s. planes) are estimated using the full covariance matrix. The cell e.s.d.'s are taken into account individually in the estimation of e.s.d.'s in distances, angles and torsion angles; correlations between e.s.d.'s in cell parameters are only used when they are defined by crystal symmetry. An approximate (isotropic) treatment of cell e.s.d.'s is used for estimating e.s.d.'s involving l.s. planes.
Refinement. Refinement of *F*^2^ against ALL reflections. The weighted *R*-factor *wR* and goodness of fit *S* are based on *F*^2^, conventional *R*-factors *R* are based on *F*, with *F* set to zero for negative *F*^2^. The threshold expression of *F*^2^ > σ(*F*^2^) is used only for calculating *R*-factors(gt) *etc*. and is not relevant to the choice of reflections for refinement. *R*-factors based on *F*^2^ are statistically about twice as large as those based on *F*, and *R*- factors based on ALL data will be even larger.

### Fractional atomic coordinates and isotropic or equivalent isotropic displacement parameters (Å^2^)

**Table:**

	*x*	*y*	*z*	*U*_iso_*/*U*_eq_	
C1	0.44525 (8)	0.57307 (12)	0.57260 (8)	0.0224 (3)	
C2	0.40060 (9)	0.51564 (12)	0.50340 (8)	0.0243 (3)	
H2	0.4079	0.4420	0.5014	0.029*	
C3	0.34516 (9)	0.56928 (13)	0.43753 (9)	0.0293 (3)	
H3	0.3152	0.5311	0.3914	0.035*	
C4	0.33398 (10)	0.67869 (14)	0.43979 (10)	0.0350 (4)	
H4	0.2970	0.7142	0.3953	0.042*	
C5	0.37828 (10)	0.73525 (13)	0.50892 (10)	0.0356 (4)	
H5	0.3707	0.8089	0.5107	0.043*	
C6	0.43372 (9)	0.68315 (12)	0.57541 (10)	0.0288 (3)	
H6	0.4631	0.7216	0.6216	0.035*	
C7	0.51617 (9)	0.41344 (12)	0.65574 (8)	0.0226 (3)	
C8	0.59225 (8)	0.40755 (12)	0.72738 (8)	0.0226 (3)	
C9	0.61904 (9)	0.51729 (12)	0.74541 (8)	0.0231 (3)	
C10	0.69450 (9)	0.56344 (13)	0.80961 (9)	0.0288 (3)	
H10A	0.7004	0.6375	0.7980	0.043*	
H10B	0.7437	0.5246	0.8116	0.043*	
H10C	0.6878	0.5578	0.8604	0.043*	
C11	0.62715 (9)	0.31029 (12)	0.75986 (8)	0.0234 (3)	
C12	0.70547 (9)	0.30187 (12)	0.82951 (9)	0.0243 (3)	
C13	0.73360 (9)	0.34085 (13)	0.90500 (9)	0.0291 (3)	
H13	0.7052	0.3870	0.9271	0.035*	
C14	0.81512 (10)	0.29751 (14)	0.94399 (9)	0.0312 (3)	
H14	0.8505	0.3098	0.9966	0.037*	
C15	0.83107 (9)	0.23514 (13)	0.88992 (9)	0.0287 (3)	
H15	0.8805	0.1970	0.8997	0.034*	
C16	0.60305 (9)	0.11363 (12)	0.75154 (9)	0.0268 (3)	
C17	0.62669 (10)	0.09001 (14)	0.83295 (10)	0.0337 (4)	
H17	0.6358	0.1454	0.8699	0.040*	
C18	0.63667 (10)	−0.01516 (15)	0.85909 (11)	0.0409 (4)	
H18	0.6530	−0.0300	0.9135	0.049*	
C19	0.62263 (10)	−0.09799 (15)	0.80517 (13)	0.0435 (5)	
H19	0.6304	−0.1685	0.8233	0.052*	
C20	0.59700 (10)	−0.07661 (14)	0.72421 (12)	0.0390 (4)	
H20	0.5864	−0.1326	0.6876	0.047*	
C21	0.58720 (9)	0.02863 (13)	0.69779 (10)	0.0302 (3)	
Cl1	0.55346 (3)	0.05471 (4)	0.59572 (3)	0.04238 (13)	
N1	0.50433 (7)	0.52104 (10)	0.63947 (7)	0.0226 (3)	
N2	0.56763 (7)	0.58347 (10)	0.69457 (7)	0.0244 (3)	
N3	0.59039 (8)	0.21931 (10)	0.72231 (7)	0.0271 (3)	
H3A	0.5546	0.2271	0.6744	0.033*	
O1	0.47138 (6)	0.33913 (8)	0.61635 (6)	0.0261 (2)	
O2	0.76468 (6)	0.23556 (8)	0.81867 (6)	0.0259 (2)	

### Atomic displacement parameters (Å^2^)

**Table:**

	*U*^11^	*U*^22^	*U*^33^	*U*^12^	*U*^13^	*U*^23^
C1	0.0186 (6)	0.0280 (7)	0.0231 (7)	0.0013 (5)	0.0104 (6)	0.0028 (6)
C2	0.0228 (7)	0.0272 (7)	0.0240 (7)	0.0006 (6)	0.0099 (6)	0.0010 (6)
C3	0.0247 (7)	0.0381 (9)	0.0241 (7)	0.0017 (6)	0.0079 (6)	0.0027 (6)
C4	0.0301 (8)	0.0403 (9)	0.0315 (8)	0.0086 (7)	0.0074 (7)	0.0095 (7)
C5	0.0354 (8)	0.0282 (8)	0.0421 (10)	0.0082 (7)	0.0127 (7)	0.0045 (7)
C6	0.0271 (7)	0.0281 (8)	0.0304 (8)	0.0012 (6)	0.0094 (6)	−0.0023 (6)
C7	0.0227 (7)	0.0265 (7)	0.0201 (7)	0.0000 (6)	0.0097 (6)	0.0009 (6)
C8	0.0215 (7)	0.0286 (7)	0.0179 (7)	−0.0010 (6)	0.0074 (5)	−0.0005 (5)
C9	0.0213 (6)	0.0289 (7)	0.0211 (7)	−0.0009 (6)	0.0102 (6)	−0.0023 (6)
C10	0.0258 (7)	0.0336 (8)	0.0243 (7)	−0.0040 (6)	0.0058 (6)	−0.0039 (6)
C11	0.0243 (7)	0.0295 (7)	0.0178 (7)	−0.0003 (6)	0.0092 (6)	−0.0005 (6)
C12	0.0237 (7)	0.0277 (7)	0.0226 (7)	0.0024 (6)	0.0099 (6)	0.0016 (6)
C13	0.0288 (7)	0.0367 (9)	0.0224 (7)	0.0039 (6)	0.0101 (6)	−0.0009 (6)
C14	0.0265 (7)	0.0437 (9)	0.0208 (7)	0.0012 (7)	0.0054 (6)	0.0031 (6)
C15	0.0210 (7)	0.0352 (8)	0.0278 (8)	0.0036 (6)	0.0062 (6)	0.0067 (6)
C16	0.0207 (7)	0.0283 (8)	0.0306 (8)	0.0015 (6)	0.0084 (6)	0.0035 (6)
C17	0.0293 (8)	0.0387 (9)	0.0292 (8)	−0.0028 (7)	0.0059 (7)	0.0058 (7)
C18	0.0289 (8)	0.0452 (10)	0.0430 (10)	−0.0031 (7)	0.0064 (7)	0.0176 (8)
C19	0.0271 (8)	0.0351 (9)	0.0668 (13)	0.0025 (7)	0.0151 (8)	0.0189 (9)
C20	0.0288 (8)	0.0295 (9)	0.0619 (12)	−0.0015 (7)	0.0203 (8)	−0.0023 (8)
C21	0.0240 (7)	0.0322 (8)	0.0355 (8)	−0.0012 (6)	0.0122 (6)	−0.0005 (7)
Cl1	0.0543 (3)	0.0414 (2)	0.0326 (2)	−0.0103 (2)	0.01723 (19)	−0.01043 (18)
N1	0.0204 (6)	0.0253 (6)	0.0206 (6)	−0.0018 (5)	0.0057 (5)	−0.0014 (5)
N2	0.0216 (6)	0.0279 (6)	0.0229 (6)	−0.0032 (5)	0.0071 (5)	−0.0039 (5)
N3	0.0291 (6)	0.0281 (7)	0.0196 (6)	0.0012 (5)	0.0034 (5)	0.0014 (5)
O1	0.0252 (5)	0.0264 (5)	0.0232 (5)	−0.0031 (4)	0.0045 (4)	−0.0001 (4)
O2	0.0243 (5)	0.0291 (5)	0.0250 (5)	0.0030 (4)	0.0097 (4)	0.0003 (4)

### Geometric parameters (Å, º)

**Table:**

C1—C6	1.391 (2)	C11—C12	1.466 (2)
C1—C2	1.395 (2)	C12—C13	1.350 (2)
C1—N1	1.4135 (18)	C12—O2	1.3746 (17)
C2—C3	1.388 (2)	C13—C14	1.420 (2)
C2—H2	0.9300	C13—H13	0.9300
C3—C4	1.381 (2)	C14—C15	1.345 (2)
C3—H3	0.9300	C14—H14	0.9300
C4—C5	1.387 (2)	C15—O2	1.3659 (18)
C4—H4	0.9300	C15—H15	0.9300
C5—C6	1.385 (2)	C16—C21	1.391 (2)
C5—H5	0.9300	C16—C17	1.396 (2)
C6—H6	0.9300	C16—N3	1.4058 (19)
C7—O1	1.2456 (17)	C17—C18	1.382 (2)
C7—N1	1.3727 (19)	C17—H17	0.9300
C7—C8	1.4602 (19)	C18—C19	1.375 (3)
C8—C11	1.384 (2)	C18—H18	0.9300
C8—C9	1.442 (2)	C19—C20	1.380 (3)
C9—N2	1.3047 (19)	C19—H19	0.9300
C9—C10	1.497 (2)	C20—C21	1.385 (2)
C10—H10A	0.9600	C20—H20	0.9300
C10—H10B	0.9600	C21—Cl1	1.7362 (17)
C10—H10C	0.9600	N1—N2	1.4060 (16)
C11—N3	1.3522 (19)	N3—H3A	0.8600
			
C6—C1—C2	120.02 (13)	C13—C12—C11	135.20 (14)
C6—C1—N1	119.33 (13)	O2—C12—C11	114.55 (12)
C2—C1—N1	120.62 (13)	C12—C13—C14	106.54 (14)
C3—C2—C1	119.50 (14)	C12—C13—H13	126.7
C3—C2—H2	120.3	C14—C13—H13	126.7
C1—C2—H2	120.3	C15—C14—C13	106.59 (14)
C4—C3—C2	120.72 (15)	C15—C14—H14	126.7
C4—C3—H3	119.6	C13—C14—H14	126.7
C2—C3—H3	119.6	C14—C15—O2	110.69 (13)
C3—C4—C5	119.49 (15)	C14—C15—H15	124.7
C3—C4—H4	120.3	O2—C15—H15	124.7
C5—C4—H4	120.3	C21—C16—C17	118.14 (15)
C6—C5—C4	120.68 (15)	C21—C16—N3	119.48 (14)
C6—C5—H5	119.7	C17—C16—N3	122.27 (14)
C4—C5—H5	119.7	C18—C17—C16	120.49 (17)
C5—C6—C1	119.59 (15)	C18—C17—H17	119.8
C5—C6—H6	120.2	C16—C17—H17	119.8
C1—C6—H6	120.2	C19—C18—C17	120.45 (17)
O1—C7—N1	126.47 (13)	C19—C18—H18	119.8
O1—C7—C8	128.96 (13)	C17—C18—H18	119.8
N1—C7—C8	104.56 (12)	C18—C19—C20	120.05 (16)
C11—C8—C9	133.20 (13)	C18—C19—H19	120.0
C11—C8—C7	121.62 (13)	C20—C19—H19	120.0
C9—C8—C7	104.97 (12)	C19—C20—C21	119.67 (17)
N2—C9—C8	111.44 (13)	C19—C20—H20	120.2
N2—C9—C10	117.86 (13)	C21—C20—H20	120.2
C8—C9—C10	130.69 (13)	C20—C21—C16	121.15 (16)
C9—C10—H10A	109.5	C20—C21—Cl1	119.34 (14)
C9—C10—H10B	109.5	C16—C21—Cl1	119.51 (12)
H10A—C10—H10B	109.5	C7—N1—N2	112.08 (11)
C9—C10—H10C	109.5	C7—N1—C1	129.33 (12)
H10A—C10—H10C	109.5	N2—N1—C1	118.15 (12)
H10B—C10—H10C	109.5	C9—N2—N1	106.94 (12)
N3—C11—C8	118.36 (13)	C11—N3—C16	128.37 (13)
N3—C11—C12	118.65 (13)	C11—N3—H3A	115.8
C8—C11—C12	122.83 (13)	C16—N3—H3A	115.8
C13—C12—O2	110.18 (13)	C15—O2—C12	106.00 (11)
			
C6—C1—C2—C3	0.3 (2)	N3—C16—C17—C18	178.52 (14)
N1—C1—C2—C3	−177.69 (12)	C16—C17—C18—C19	−0.6 (2)
C1—C2—C3—C4	0.2 (2)	C17—C18—C19—C20	−1.1 (3)
C2—C3—C4—C5	−0.5 (2)	C18—C19—C20—C21	1.3 (2)
C3—C4—C5—C6	0.2 (2)	C19—C20—C21—C16	0.2 (2)
C4—C5—C6—C1	0.3 (2)	C19—C20—C21—Cl1	−179.07 (12)
C2—C1—C6—C5	−0.6 (2)	C17—C16—C21—C20	−1.9 (2)
N1—C1—C6—C5	177.51 (13)	N3—C16—C21—C20	−178.43 (14)
O1—C7—C8—C11	2.7 (2)	C17—C16—C21—Cl1	177.36 (11)
N1—C7—C8—C11	−176.44 (12)	N3—C16—C21—Cl1	0.88 (19)
O1—C7—C8—C9	178.06 (14)	O1—C7—N1—N2	−178.37 (12)
N1—C7—C8—C9	−1.11 (14)	C8—C7—N1—N2	0.82 (15)
C11—C8—C9—N2	175.64 (15)	O1—C7—N1—C1	−6.2 (2)
C7—C8—C9—N2	1.09 (16)	C8—C7—N1—C1	173.00 (12)
C11—C8—C9—C10	−2.8 (3)	C6—C1—N1—C7	167.80 (14)
C7—C8—C9—C10	−177.36 (14)	C2—C1—N1—C7	−14.1 (2)
C9—C8—C11—N3	−171.97 (14)	C6—C1—N1—N2	−20.42 (18)
C7—C8—C11—N3	1.8 (2)	C2—C1—N1—N2	157.62 (12)
C9—C8—C11—C12	3.4 (2)	C8—C9—N2—N1	−0.60 (15)
C7—C8—C11—C12	177.17 (12)	C10—C9—N2—N1	178.07 (11)
N3—C11—C12—C13	−129.37 (19)	C7—N1—N2—C9	−0.17 (15)
C8—C11—C12—C13	55.3 (2)	C1—N1—N2—C9	−173.31 (11)
N3—C11—C12—O2	47.33 (18)	C8—C11—N3—C16	−165.70 (14)
C8—C11—C12—O2	−127.98 (14)	C12—C11—N3—C16	18.8 (2)
O2—C12—C13—C14	0.34 (18)	C21—C16—N3—C11	−154.71 (15)
C11—C12—C13—C14	177.15 (16)	C17—C16—N3—C11	29.0 (2)
C12—C13—C14—C15	−0.11 (19)	C14—C15—O2—C12	0.37 (17)
C13—C14—C15—O2	−0.17 (18)	C13—C12—O2—C15	−0.44 (16)
C21—C16—C17—C18	2.1 (2)	C11—C12—O2—C15	−177.97 (12)

### Hydrogen-bond geometry (Å, º)

**Table:**

*D*—H···*A*	*D*—H	H···*A*	*D*···*A*	*D*—H···*A*
N3—H3*A*···O1	0.86	2.00	2.678 (2)	135
C15—H15···N2^i^	0.93	2.59	3.282 (2)	131

Symmetry code: (i) −*x*+3/2, *y*−1/2, −*z*+3/2.
