# Supplementary figures and images for: Crystal structure of (4Z)-4-{[(2-chloro­phen­yl)amino](furan-2-yl)methyl­idene}-3-methyl-1-phenyl-4,5-di­hydro-1H-pyrazol-5-one
Source: Acta Crystallogr E Crystallogr Commun. 2015 Feb 13;71(Pt 3):o177–8. doi: 10.1107/S2056989015002698 (PMC4350689; doi:10.1107/S2056989015002698)

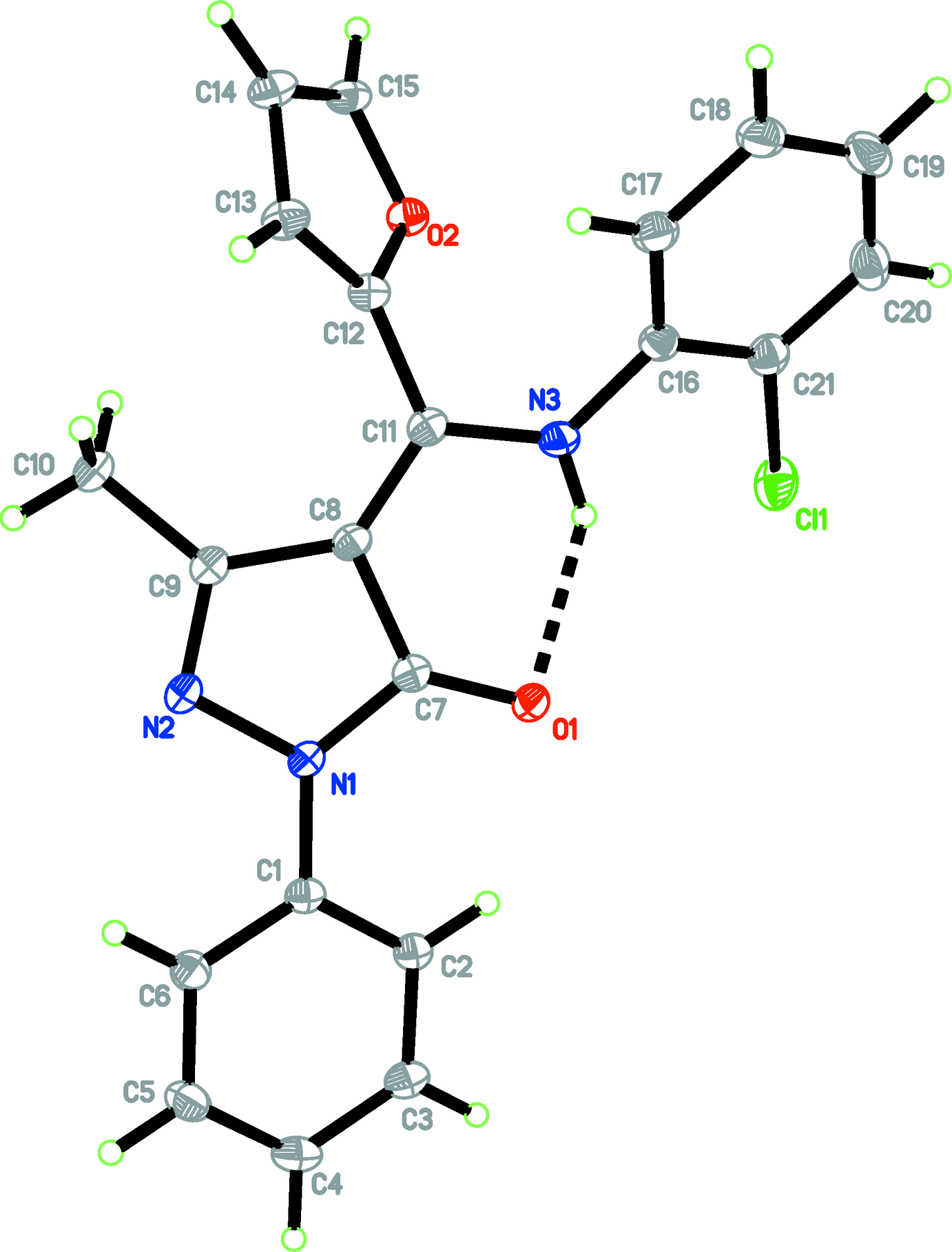

Supplement: Supplementary file 4 [file e-71-0o177-fig1.tif]

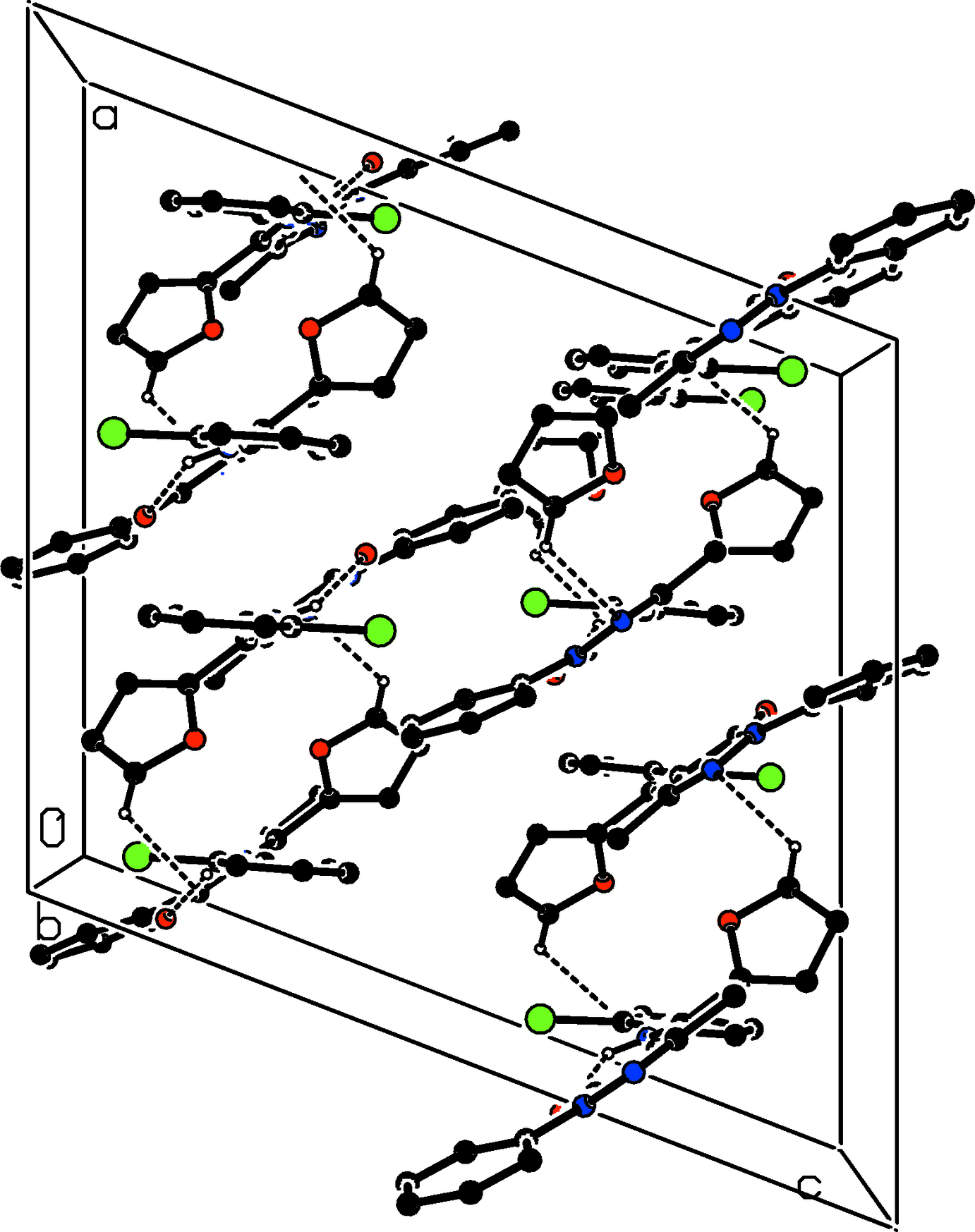

Supplement: Supplementary file 5 [file e-71-0o177-fig2.tif]
